# Synthesis of Silver Nanoparticles from Bitter Melon (*Momordica charantia*) Extracts and Their Antibacterial Effect

**DOI:** 10.3390/microorganisms13081809

**Published:** 2025-08-02

**Authors:** Nanh Lovanh, Getahun Agga, Graciela Ruiz-Aguilar, John Loughrin, Karamat Sistani

**Affiliations:** 1 Food Animal Environmental Systems Research Unit, Agricultural Research Service, U.S. Department of Agriculture, Bowling Green, KY 42101, USA; anajucklao@gmail.com (N.L.); john.loughrin@usda.gov (J.L.); karamat.sistani@usda.gov (K.S.); 2 Department of Environmental Sciences, Division of Life Sciences, University of Guanajuato, Irapuato 36500, Guanajuato, Mexico; gracielar@ugto.mx

**Keywords:** silver nanoparticles, optimization, green synthesis, pathogens, antimicrobial resistance

## Abstract

We utilized silver nanoparticles synthesized from bitter melon (*Momordica charantia*) extracts for testing against the common agricultural pathogen *Escherichia coli*. The synthesized nanoparticles were characterized and confirmed as silver nanoparticles by using ultraviolet spectroscopy, Fourier transform infrared spectroscopy, and scanning electron microscopy analysis. The results show that AgNPs were effective against *E. coli* ATCC25922 strain. The AgNPs had an increased potency against the *E. coli* strain in optimum culture media compared to silver ions alone. AgNP-treated cultures achieved a kill percentage of 100% in less incubation time and at a lower dosage than those treated with silver ions alone. The powder form of the AgNPs also showed remarkable potency against *E. coli* in solution. Based on these findings, the current method is suitable for the industrial-scale production of AgNPs from a commonly available edible plant with known medicinal benefits in the fight against foodborne pathogens, including antibiotic-resistant strains.

## 1. Introduction

The United Nations [1] has recognized antimicrobial resistance (AMR) as one of the most urgent global health threats and that it requires a One Health response that involves the integration of actions between humans, animals (and plants), and the environment [2,3]. AMR-related mortality, particularly in people older than 5 years of age, in all regions of the world has increased over the past 31 years [4]. The rise in antibiotic-resistant microorganisms is mainly attributed to the increasing use and, at times, inappropriate use of antibiotics in human medicine and animal production [5]. Globally, most of the antibiotics used in livestock production are given for growth promotion and as prophylactic agents, in addition to treating bacterial infections [6]. These practices increase the selection of antibiotic resistance in microbial populations. Antibiotic-resistant microorganisms may then be transmitted to humans through human-to-human transmission, through the food chain, or through the environment, where these microorganisms can cause diseases that may not be treated by available antibiotics [7]. Therefore, it is necessary to find a simple and economical way to reduce the proliferation of antibiotic-resistant microorganisms, such as an alternative to antibiotics, particularly for use in food animal production [8,9]. Along with medicinal plants, nanoparticles with antimicrobial properties, especially nanoparticles that can be synthesized by eco-friendly methods, hold great promise in this area [10,11,12]. Controlling foodborne pathogens, including antimicrobial-resistant strains, in food animal production facilities through the use of nanotechnology will ultimately contribute to the fight against antimicrobial-resistant bacteria, which is of public health importance [11]. The utilization of an optimized protocol at the farm level lies at the intersection of the environment and the public, contributing to the One Health approach of fighting against antimicrobial resistance.

Earlier reports described nanoparticles that are synthesized through various techniques like electrochemical methods, aerosol technologies, ultraviolet irradiation, photochemical methods, the use of ultrasonic fields, and laser ablation techniques. However, these previous studies used hazardous chemicals and consumed high levels of energy for low levels of material conversion [12,13,14]. In the current environment, interest in the synthesis of nanoparticles, especially silver nanoparticles (AgNPs), through efficient alternative and eco-friendly methods such as green chemistry and the use of biocompatible compounds such as plant, bacterial, and fungal extracts and enzymes has gained momentum [10,11,12,15]. However, plants are the best candidates for large-scale biosynthesis of metallic NPs, primarily due to the occurrence of secondary metabolites, also conferring higher stability and a faster rate of production of NPs than do microorganisms [16]. Furthermore, vegetable waste from food processing industries can be directly used for this purpose, providing a cost-effective means of metallic NP production and waste management, and thereby providing a cleaner environment for the public [16]. In recent years, the AgNP field of research has been focusing on the extracts from various parts of plants (leaves, fruits, seeds, latex, and peel); in particular, leaf-mediated synthesis has been widely studied, for example, in *Alternanthera dentate* [17], *Abutilon indicum* [18], *Ziziphora tenuior* [19], *Ficus carica* [20], *Cymbopogan citrates* [21], *Acalypha indica* [22], *Premna herbacea* [23], *Centella asiatica* [24], *Brassica rapa* [25], *Coccinia indica* [26], *Vitex negundo* [27], *Melia dubia* [28], *Portulaca oleracea* [29], *Pogostemon benghalensis* [30], *Swietenia mahogany* [31], *Moringa oleifera* [32], *Garcinia mangostana* [33], *Eclipta prostrate* [34], *Helianthus tuberosus* [35], *Solanum nigrum*, *Solanum indicum* [36,37], and *Nelumbo nucifera* [38]. The rationale behind these studies is that plant extracts can be used in a silver solution, e.g., silver nitrate (AgNO_3_), which, when heated with the plant extract, reduces and stabilizes the silver ion to Ag^0^ with or without encapsulation using an additional delivery mechanism such as silica [39,40].

Nanomaterials are applied in various domains, such as cosmetics, biomedical, food packaging, drug gene delivery, environmental, optics, chemical, electronics, space, energy science, photo electrochemistry, and catalysis applications [11,41,42]. In agriculture, they are used as nanofertilizers to improve the efficiency of nutrient utilization and as nanopesticides for the control of fungal and bacterial infections in plants [43]. Furthermore, the vast potential of nanoparticles has been explored in animal production and food safety as alternatives to antibiotics for growth promotion and to control animal pathogens such as pathogenic *E. coli* and *Staphylococcus aureus* [44]. In general, nanomaterials are also used in antimicrobial and pathogen reduction and management in livestock production, which are the main topics of this paper. The application is dependent on the size (1 to 100 nm) and shape (spherical, circular, hexagonal, etc.) of the nanoparticles [14]. In particular, platinum, gold, and silver nanoparticles are recognized as noble metals and are significantly applied in magnetic, optoelectronics, electronics, and information storage applications [45]. Among the noble metals, AgNPs hold a prominent place due to their unique properties, such as good conductivity, chemical stability, catalytic aspects, and—most importantly—pharmacological aspects, such as antibacterial, anti-fungal, antiviral, and anti-inflammatory activities [12,46].

Silver nanoparticles have been shown to have antimicrobial activities [10]. AgNPs have been used in antimicrobial consumer products in the health, textile, cosmetic, appliance, environmental, and construction sectors [15]. A few of the health applications approved by the U.S. Food and Drug Administration include wound healing, facial masks, textile fibers, sanitizers, coatings on surgical tools, dental implants, and urinary catheters [15]. The top three sectors, the medical, textile, and cosmetic-related products, are the most prominent use categories, making up ~55% of all products containing nanoparticles [15]. The agriculture sector consumes less than two percent of silver nanoparticles [15], indicating a vast potential for application in food animal production and food safety. AgNPs and Ag^+^ ions have gained increased attention, particularly due to their potential use in the fight against two major health threats, AMR and viral infections, where treatment options are either limited or absent [15]. Since AgNPs have multiple mechanisms of action, as opposed to a single mechanism of action exhibited by the antibiotics or antiviral agents, the development of resistance to AgNPs is less likely when compared to specific antiviral or antibiotic therapies [15]. Thus, the development of novel applications of AgNPs makes them a unique alternative to antibiotics and allows them to serve as inactivation agents against antibiotic-resistant microorganisms.

The antimicrobial effects of AgNPs synthesized from bitter melon extracts have been tested against diverse microbes, including bacteria, fungi, and helminths [11,47,48,49,50]. However, previous studies presented mainly qualitative data regarding minimum inhibitory concentration (MIC), reporting only the presence or absence of bacterial growth. The aim of the current study was to provide more quantitative data, the exposure time needed and the percent kill of the AgNPs at varying concentrations. To achieve this objective, AgNPs were synthesized from bitter melon extract, a known medicinal plant with antioxidant properties [11,51], as a model to utilize and examine the effectiveness of AgNPs against bacterial pathogens. Our study provides a protocol that can be utilized in situ at food animal production facilities for potential pathogen reduction after rigorous risk assessments with regard to its safety and toxicity [16].

## 2. Materials and Methods

### 2.1. Preparation of Bitter Melon Extract 

Whole bitter melons (*Momordica charantia*) were collected from the local garden (Bowling Green, KY, USA) and washed several times in distilled water to remove dirt. One hundred grams of bitter melon was cut into small pieces and heated in 750 mL of nanopure water in a 1000 mL beaker for 30 min at 90 °C to obtain the melon extract, followed by filtration using Whatman No. 42 (Sigma-Aldrich, Inc., St. Louis, MO, USA) and storage at 4 °C for future experiments.

### 2.2. Synthesization of Silver Nanoparticles

Production of AgNPs was optimized under various reaction conditions including variations in pH (3, 4, 5, 6, 7, 8, 9, and 10), melon extract reaction volume (0.5, 1, 1.5, 2, 2.5, 3, 3.5, 4, 4.5, and 5 mL), silver ion solution concentration (0.1, 0.2, 0.3, 0.4, 0.5, 0.6, 0.7, 0.8, 0.9, and 1.0 mM), and time (0, 15, 30, 45, 60, 75, 90, 105, 120, 135, 150, 165, 180, 195, 210, 240, and 270 min). While each parameter was optimized, the other parameters remained stable, following a previously published protocol [52]. For this study, the optimum conditions of alkaline pH of 9 and 9 mL of bitter melon extract mixed with 2 mM silver nitrate solution were utilized to obtain AgNPs. The pH readings were adjusted by using hydrochloric acid and potassium hydroxide for acidic and alkaline pHs, respectively. For synthesis of AgNPs using the melon extract, 2 mL of 50 mM silver nitrate stock solution was added to 48 mL of bitter melon extract to obtain a final concentration of 2 mM silver nitrate. The extract was added to 100 mL Erlenmeyer flasks, and the reactions were carried out under ambient conditions. The reaction mixture was observed for a change in color as validation of AgNP synthesis. The intensity of color was recorded between 200 and 800 nm on an ultraviolet–visible spectrometer (UV–1800 UV–Vis spectrophotometer, Shimadzu, Kyoto, Japan). After a reaction time of 24 h, the solution was centrifuged, and lyophilization was carried out to obtain the powder of the AgNPs.

### 2.3. Separation of Silver Nanoparticles

The AgNPs were isolated from the optimized mixture as follows: The obtained reaction mixture was subjected to centrifugation at 12,000 rpm for 10 min; the pellet was purified using nanopure water (Sigma-Aldrich, Inc.) and washed repeatedly to ensure better separation of free entities from the AgNPs. The obtained AgNPs were lyophilized and used for further characterization and assessment of their antimicrobial potential.

### 2.4. Spectral and Microscopic Analyses of Silver Nanoparticles

Fourier transform infrared spectroscopy (Perkin–Elmer FTIR spectrophotometer, Norwalk, CT, USA) spectra of the AgNPs were recorded in the diffuse reflectance mode at a resolution of 4 particles cm^−1^ in KBr pellets. X-ray diffraction (XRD) analysis of AgNPs was carried out on a Rigaku instrument (Rigaku Americas, St. Joseph, MI, USA) at 40 kV and 30 mA using CuKα radiation with a wavelength of 1.5406 Å and a nickel monochromator filtering wave operating at a tube voltage of 40 kV and a tube current of 30 mA. The scanning was performed in the 2θ range of 5–90° at 0.04°/min with a time constant of 2 s. Further, the morphologies, crystalline natures, and size distributions of the AgNPs were analyzed using a high-resolution transmission electron microscopy model (JEOL–2010, Akishima, Tokyo, Japan).

### 2.5. Testing of Silver Nanoparticles for Their Antimicrobial Activities

The antimicrobial activities of metallic silver, melon extract, AgNP solutions, and powdered AgNPs were evaluated using a AgNP mixture (NPM) assay against *E. coli* ATCC25922 (American Type Culture Collection). The strain was maintained on tryptic soy agar (TSA; Becton Dickinson and Company, Franklin Lakes, NJ, USA) and cultured in tryptic soy broth (TSB), which was determined to provide sufficient nutrient availability compared to minimal medium (M9; Thermo Fisher Scientific, Waltham, MA, USA) or no nutrients (sterile water). For each assay, *E. coli* was grown to an initial inoculum concentration of ~1 × 10^9^ colony forming units (CFUs)/mL by inoculating a single colony into 50 mL of TSB, followed by incubation at 37 °C with shaking (150 rpm) for 24 h.

An NPM suspension of various concentrations of silver, AgNP solutions, and powdered AgNPs was prepared with TSB and 1 mL of *E. coli* inoculum. The TSB cultures were incubated at 37 °C. CFU counts were determined from TSB culture at 0, 2, 4, and 24 h post-treatment. CFU concentration was calculated based on the optical density from a previously determined standard curve [53]. To determine *E. coli* concentrations, 100 µL aliquots at each incubation time were serially diluted and spread plated onto TSA plates by using an Eddy Jet 2 spiral plater (Neutec Group Inc., Farmingdale, NY, USA), incubated at 37 °C, and counted by using a Sphere-Flash automated colony counter (Neutec Group, Inc.).

Percent kill was calculated as follows:

(1) $$Kill\;Percentage=\frac{Ci-Ct}{Ci}\;\times 100$$

where C_i_ is the initial CFU count and C_t_ is the CFU count at time t.

## 3. Results and Discussion

### 3.1. Characterization of Silver Nanoparticles

When the *Momordica charantia* extract was mixed with silver nitrate in aqueous solution, the color changed from yellowish to dark purple to brownish over time. The change in color was due to the reduction of Ag^+^ ions to Ag^0^ nanoparticles [33,34,54,55]. In our study, the AgNP solution was a dark brown colored colloidal solution, which was due to the extracellular synthesis of AgNPs on the addition of the aqueous silver nitrate solution to the bitter melon extract. The change in color from a weakly colored watery solution to brown occurs due to the surface plasmon resonance (SPR) phenomenon of metal nanoparticles in the reaction mixture [12,17,31,41]. An earlier report suggests that biomolecules such as fatty acids, alcohols, phytosterols, higher terpenoids, flavonoids, -O- and -C-glycosides, and phenolic acids present in the extract [11,12,56] play a vital role in the conversion of the ionic form of silver to the metallic nanoparticles. In our study, since the bitter melon is enriched with polyphenols, glycosides, saponins, alkaloids, and free acids [12,57,58], we anticipated that these phytochemicals with their hydroxyl moieties would act as reducing agents and promote the reduction of Ag^+^ ions to zero-valent Ag atoms [11,12,59].

**Figure 1:**
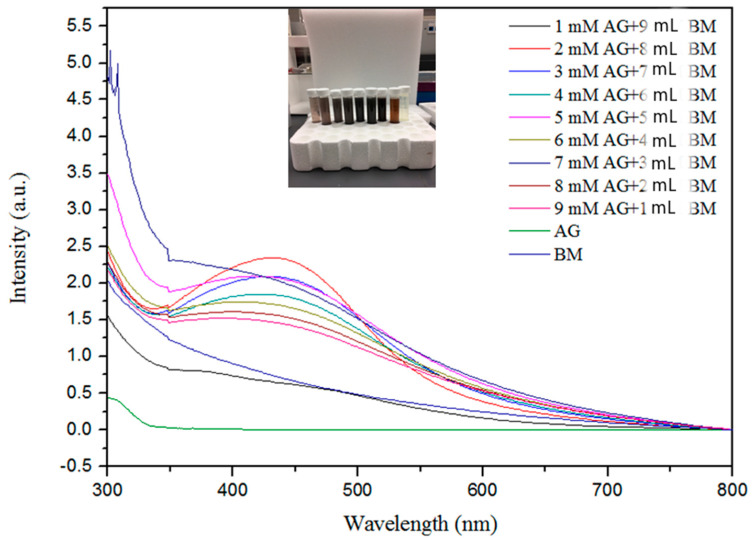
Ultraviolet–visible spectroscopy absorption spectra expressed as absorbance unit (a.u.) of silver nanoparticles with different concentrations of Ag^+^ metal ions from 1.0 to 9.0 mM and bitter melon extract (BM).

The formation of AgNPs was observed using UV–visible spectroscopy. Figure 1 represents the UV–visible spectra of AgNPs with absorption maxima in the range of 420 nm to 450 nm, which are in agreement with previous findings [12,38,54,60,61]. The shift in absorbance from standard 430 nm may be attributed to the capping of AgNPs by the bioactive constituents in the bitter melon extract [57,62]. The single broad SPR peak in the spectra is likely due to the spherical nature of the AgNPs and their dispersion in the solution [45].

Figure 1 shows the effect of different volumes of the bitter melon extract and concentrations of AgNO_3_ on the synthesis of AgNPs. The absorption band rises under increasing reaction volume of bitter melon extract on addition of silver nitrate solution, exhibiting analogous association up to 9 mL, indicating a higher amount of silver ion reduction, whereas supplementation of higher molarities of silver ions with smaller volumes of melon extracts contrarily delimits the reduction as indicated by decreased peak absorbance. This contrary relationship exemplifies the constraint that exists in reaction due to a higher volume of extract reacting with a precise volume of silver nitrate solution in the reaction mixture [63].

The maximum absorption was attained at 2.0 mM silver nitrate solution, as shown in Figure 1, indicating much higher oxidation of hydroxyl groups by the monovalent silver ions. The peak absorbance persistently increased with a decrease in concentration from 9 mM to 2 mM, illustrating the parallel relationship with the metal ion concentration. It appeared that with higher concentrations of melon extract for capping and reduction of silver ions to neutral silver, more AgNPs were formed, as indicated by the higher peak on the UV-Vis spectrum. The increase in intensity with an increase in extract volumes may be attributed to capping of AgNPs with phytochemicals [64].

**Figure 2:**
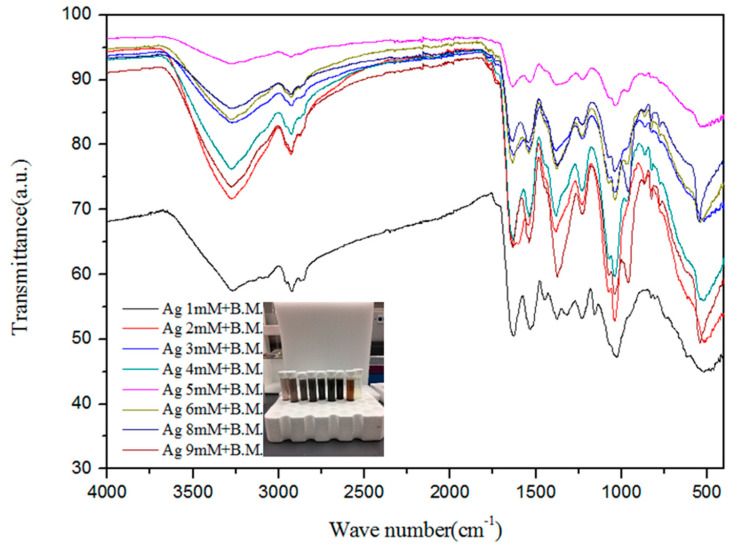
Fourier transform infrared spectra expressed as absorbance units (a.u.) of silver nanoparticles synthesized from different concentrations of silver ions and bitter melon (B.M.).

The Fourier transform infrared (FTIR) spectra of the extract and AgNPs were recorded to gain insights into the nature of the biomolecules responsible for the reduction of Ag^+^ ions (Figure 2). The absorption spectra of AgNPs at various concentrations indicate the involvement of various biomolecules such as proteins and phenols in the reduction of Ag^+^ ions to Ag atoms. The absorption peak at 3260 cm^−1^ is due to the N–H stretch vibrations of primary amines, and the strong peak at 2931 cm^−1^ corresponds to the stretching of O–H of carboxylic acids and their derivatives. The strong peak at 1614 cm^−1^ occurs due to –NH_2_ bending vibrations of aliphatic amines. The band existence at 1383 cm^−1^ is due to the in-plane bending vibrations of the –O–H group. The peak 1240 cm^−1^ corresponds to the C–N stretching vibration of aliphatic amines [65]. The strong peak at 1078 cm^−1^ is attributed to C–O stretching, possibly due to the presence of carboxylic acid groups. Thus, the presence of –NH, -OH, and C–O confirms the presence of proteins and carboxylic acid derivatives, as well as phenols, in the extract. The peaks were similar for the various concentrations of silver nitrate reacted with melon extracts.

**Figure 3:**
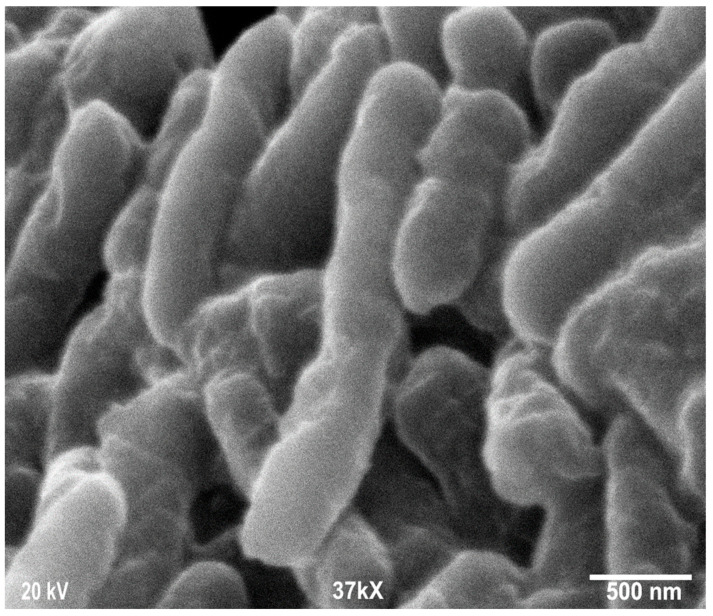
Scanning electron microscopy images of silver nanoparticles (AgNPs) synthesized from bitter melon extracts showing rod-like morphology.

**Figure 4:**
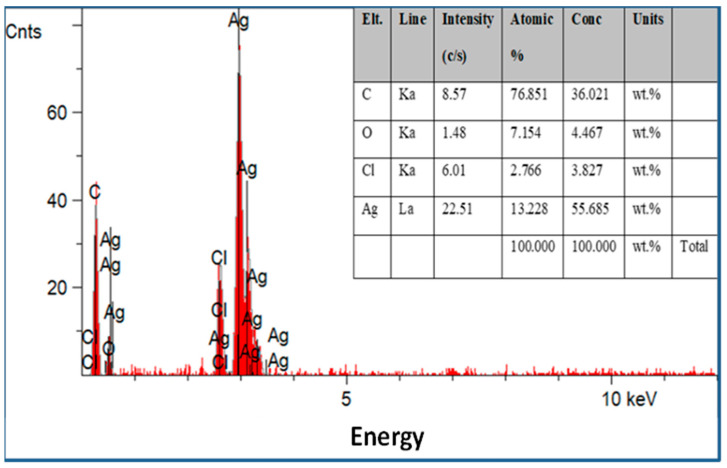
Energy-dispersive X-ray spectroscopy patterns of the silver nanoparticles synthesized with optimum parameters by bitter melon extract with other elements presented.

Scanning electron microscopy was employed to determine the morphology, size, and shape of the nanoparticles. The shape of the larger nanoparticles was rod-like, with an average length of ~20 to 70 nm and with aggregation (Figure 3). SEM micrographs provided further insight into the morphology and particle size distribution profile of the bitter melon extract AgNPs. It was observed that AgNPs with the smallest size distribution were spherical and had a size range of 14.5 ± 8.2 nm. To confirm the crystallinity of AgNPs, the selected area electron diffraction (SAED) patterns recorded single particles in the aggregates of all the nanoparticle samples corresponding to a characteristic polycrystalline ring pattern for a face-centred-cubic structure, revealing that the synthesized AgNPs were crystalline in nature. Furthermore, the crystalline nature of AgNPs agrees with the X-ray diffraction pattern [42]. The energy-dispersive X-ray diffraction spectroscopy (EDX) pattern depicts the crystallinity of AgNPs and the oxide pattern as shown in Figure 4. It is apparent that characteristic absorption peaks of Ag occurred around 3 keV, which is similar to an earlier study [66]. This absorption peak is typical for metallic silver nanocrystallites due to surface plasmon resonance. There are other elements presented as well here, which are carbon (36% by weight), oxygen (4.5%), and Cl^−^ ions (3.8%). The presence of these other elements helps the formation of AgNPs, as observed and corroborated well in other studies [67,68].

### 3.2. Antimicrobial Activity

**Figure 5:**
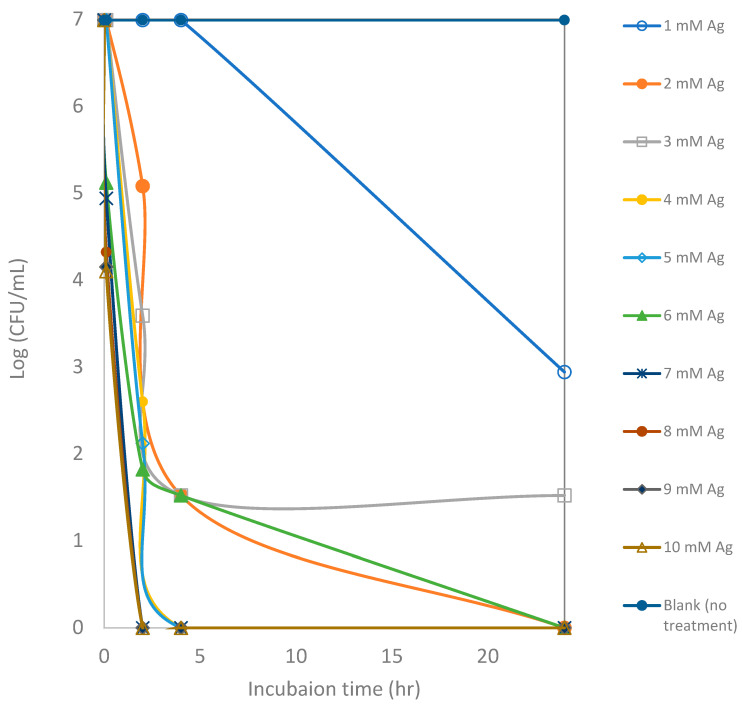
Counts of viable colony forming units (CFUs) of *E. coli* after exposure to silver ions at various concentrations for a 24 h incubation period. Each point represents an average of triplicate measurements.

**Figure 6:**
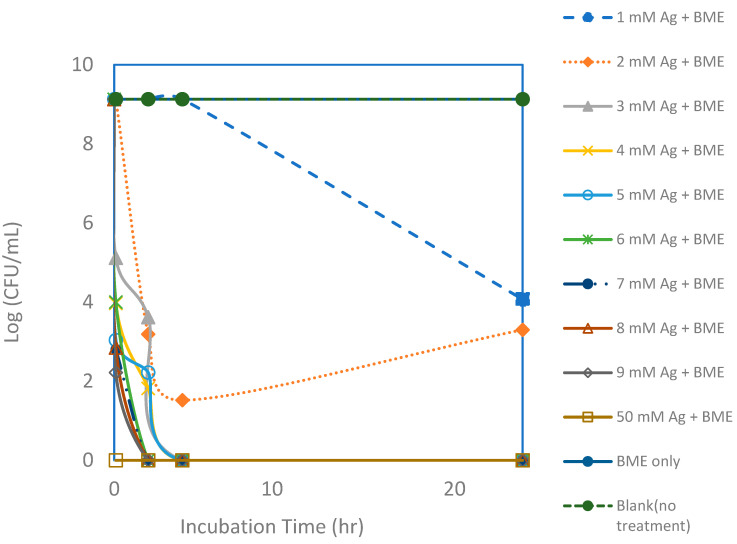
Viable colony forming unit (CFU) counts of *E. coli* after exposure to silver nanoparticle (AgNP) and bitter melon extract (BME) solutions with various silver concentrations for a 24 h incubation period. Each point represents an average of triplicate measurements.

Silver is used in multiple consumer products and biomedical applications due to its antimicrobial effects [10]. However, the effectiveness of the ionic form of silver is dependent on environmental conditions such as pH, ionic strength, and the presence of organic matter [69]. In this study, the in vitro antimicrobial activity of synthesized AgNPs in comparison to the pure silver ions in the form of silver nitrate was carried out against *E. coli* as a model bacterium. AgNPs synthesized from bitter melon extract and silver ions showed remarkable activity against *E. coli* in culture medium (Figure 5 and Figure 6). Figure 5 shows the effectiveness of different concentrations of silver ions (silver nitrate) against *E. coli* in TSB medium. It appears that the lower concentrations of silver ions (1 to 3 mM) take a longer time to inactivate an *E. coli* culture of about 1.0 × 10^7^ CFUs per mL (or 7 logs). After 24 h of incubation, the CFU counts remain between 1 log and 3 logs for assays with 1 to 3 mM silver ions. Total inactivation was achieved with higher Ag ion concentrations of 6 mM or higher. Total killing at initial exposure was achieved with the assays that contained 6 mM or higher concentrations of Ag ions.

**Figure 7:**
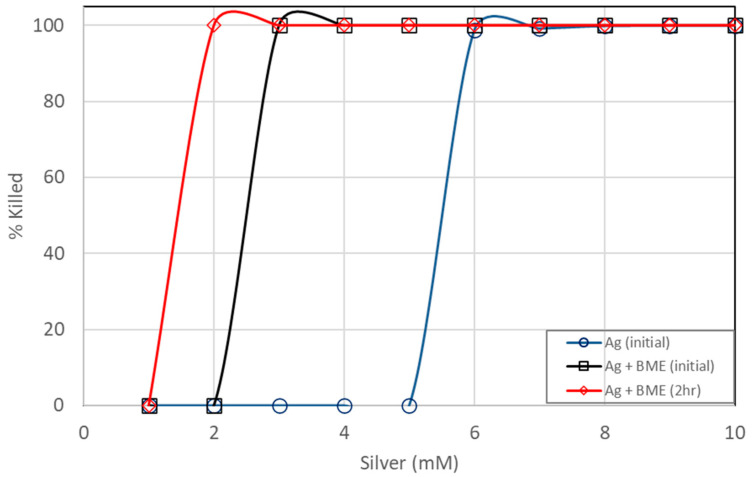
Comparison of *E. coli* kill percentages between pure silver ions and silver nanoparticles prepared from bitter melon extracts.

Even though inactivation was readily achieved with just silver ions, it appears that inactivation could be achieved faster and using lower dosages with the added bitter melon extracts, as shown in Figure 6 and Figure 7. Total killing of *E. coli* was achieved with the assay containing 3 mM of Ag^+^ reacting with bitter melon extract. Even with a higher CFU concentration of an initial concentration of about 1 × 10^9^ CFUs per mL, 100% kill was achieved with about 3 mM of Ag ions when bitter melon extract was added. Bitter melon extracts alone and blank (no treatment) assays, on the other hand, showed no reduction in *E. coli* counts. Figure 7 shows a comparison of the effectiveness of Ag ions versus the AgNPs in vitro against *E. coli*. Total kill was achieved quickly and at a lower dosage with AgNPs in solution than with Ag ions alone. It took about 3 mM less silver ions when bitter melon extract was added to obtain 100% kill at 0 hr incubation. It took fewer silver ions (2 mM) for total kill after two hours of incubation with bitter melon extract added.

The present study was conducted with a linear increase in the concentrations (0–9 mM) of silver ions or with AgNPs in conjunction with bitter melon. Therefore, it is not possible to directly compare results with previous studies [47] that evaluated the antimicrobial impacts of bitter-melon-based AgNPs by using the standard broth microdilution method to determine their minimum inhibitory concentration. Broth microdilution involves the use of a specific broth medium, such as Mueller–Hinton broth, containing geometrically increasing concentrations (typically a two-fold dilution series) of the antimicrobial agent, which is inoculated with a defined number of bacterial cells [70]. Since our results were read by plating the culture broth onto agar media, they provided quantitative measurements in terms of CFU/mL and percent kill as opposed to qualitative outputs of the presence or absence of bacterial growth at a given antibacterial concentration normally obtained from standard antimicrobial susceptibility testing. Furthermore, our study provided the required time for bacterial exposure to the AgNPs. Nevertheless, 3 mM can be considered the minimum inhibitory concentration (MIC) of silver ions in bitter melon. Using the powdered bitter-melon-based AgNPs (see below), we used a two-fold serial dilution usually used for broth microdilution, and 5 mg/L is the MIC that completely inhibited *E. coli* growth. An MIC value of 4 μg/mL of bitter-melon-based AgNPs was reported for *E. coli* [47].

**Figure 8:**
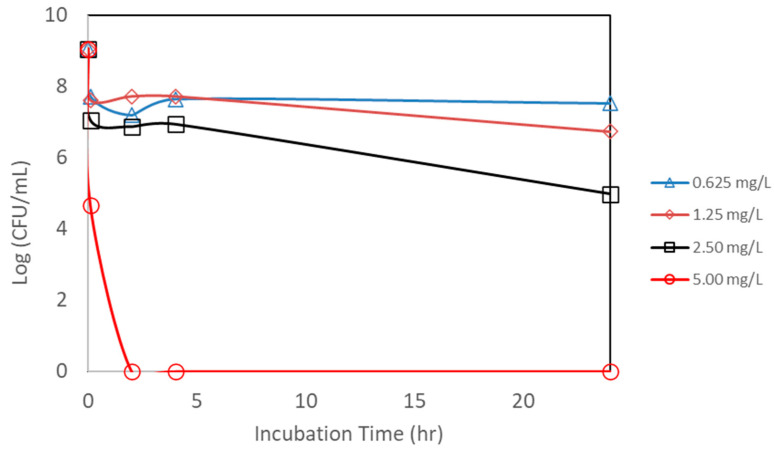
Counts of viable colony forming units (CFUs) of *E. coli* after exposure to different concentrations of nanoparticle powder. Each point represents an average of triplicate measurements.

An additional study was also conducted to see if the powdered form of AgNP has the same effectiveness against *E. coli*. AgNPs synthesized from bitter melon extract were obtained via lyophilization (drying under vacuum) to obtain the powder form for better handling and storage. A scanning electron microscopic image of the powdered form of the AgNPs is shown in Figure 3. The same bacterial assay setup was carried out as for the liquid form, except the powder form of AgNPs was used. Figure 8 shows the CFU concentration (in log units) versus the incubation time with different amounts of AgNPs (total mass of AgNPs) added. The initial *E. coli* concentration was about 1.1 × 10^9^ CFUs per mL. The lower concentrations of AgNPs (0.625 to 2.50 mg/L) could only achieve a CFU reduction of about 1 to 3 log units, even after an incubation period of 24 h. The trends are that lower concentrations achieve smaller CFU reductions, and a longer incubation time is required to achieve a higher CFU reduction. In terms of kill percentage, the lower concentrations of AgNPs were able to achieve a kill of up to 99.99%. However, it took a longer incubation time to achieve this level of kill for the lower concentrations of AgNPs. For the higher concentration of AgNPs (at 5.00 mg/L), on the other hand, total kill was achieved and obtained at a shorter incubation time. Total kill was achieved instantaneously at 0 hr incubation. Table 1 shows the kill percentages for different AgNP concentrations and incubation times.

However, we note that the above comparisons between the silver ions and aqueous and powdered forms of AgNPs with respect to bacterial actions are based on qualitative observations in the form of graphs and tables. Formal statistical comparison would require more experimental replicates to provide rigorous proof.

The value of using green technology based on a widely available crop will enable industrial-scale production of AgNPs. Green technology reduces cellular toxicity to the host (human or animal) and reduces environmental impacts. This makes it a sustainable approach for harnessing naturally available products to combat multidrug resistant bacteria threatening human and animal health. However, a comprehensive comparative evaluation of yield, energy consumption, and cost-efficiency should be performed between the proposed green synthesis methods and conventional chemical or physical nanoparticle synthesis techniques. This may require systematic reviews, meta-analyses, economic analyses, and quantitative risk assessments. Nevertheless, Kaiser et al. [15] clearly provided the advantages and the disadvantages of chemical, biological, and physical methods of AgNP synthesis. The biological method stands in the middle with respect to its cost of production, which is mainly associated with purification. Its main advantage lies in its eco-friendly and biocompatible properties.

Although we did not determine the mechanisms underlying the antibacterial effect of our AgNPs, the literature shows that the mechanisms responsible for their antibiotic properties make them an attractive alternative or adjunct to traditional antibiotics. The effectiveness of nanoparticles relies on various mechanisms rather than relying on a single mechanism, as is common with antibiotics. This makes bacterial resistance to them less likely, and the delivery of them in silica nanoparticles, as performed in this study, enhances their interaction with bacterial cells relative to transition metals given no provision for solubilization. In general, AgNPs and Ag^+^ ions, in addition to other transition metals such as Mn, Fe, Cu, Au, and Zn, cause bacterial cell membrane and DNA damage [15,71,72].

The interaction between AgNPs and bacterial cell membranes can cause physical damage through direct membrane contact, altered permeability, osmotic collapse, and leakage of K+ ions and other intracellular contents, halting cellular respiration by eliminating membrane potentials. The membrane damage in turn facilitates the entry of other noxious substances such as toxins and antibiotics and the release of reactive oxygen species (ROS), which will have a detrimental effect on bacteria [15]. DNA damage can be caused by AgNP-DNA interactions that lead to DNA denaturation, DNA breaks, mutations in the DNA repair genes, interference with cell division, and the presence of ROS [15]. Using plant extracts to synthesize metal nanoparticles adds the benefits of reducing the oxidation state of the metal, stabilizing the nanoparticles, and preventing their aggregation [73]. In addition, using plant extracts to prepare nanoparticles seems to increase their efficacy compared to nanoparticles synthesized by chemical methods [74]. Although the reason for this is not clear, it could be speculated that this may be due to the pharmacological properties of secondary metabolites in the extracts, the scavenging of free radicals by organic matter in the extracts, or simply the superior physical characteristics of the nanoparticles.

## 4. Conclusions

In the present study, we demonstrated a novel eco-friendly, rapid, and low-cost method for the synthesis of AgNPs in a green chemistry approach by bitter melon extracts to make use of a widely available medicinal and edible plant to fight against potential agricultural pathogens while avoiding the use of any toxic chemicals. As an alternative, the bioactive molecules from the melon extract serve as both reducing and capping agents. The synthesized AgNPs were characterized by UV-Vis spectroscopy, FTIR, SEM, and XRD techniques. The obtained nanoparticles presented almost rod-like shapes in sizes ranging from ~10 to 70 nm, with some aggregation. The antimicrobial activity of the AgNPs was investigated against a model agricultural pathogen, *E. coli*, and good activity was shown. The synthesized AgNPs have increased potency against *E. coli* in optimum culture media in comparison to Ag ions alone. The powder also showed a remarkable potency against *E. coli* in solution. Based on these findings, the current method can be evaluated for production yield and reproducibility suitable for the industrial-scale production of AgNPs from a commonly available edible plant in a fight against agricultural pathogens that could harbor antibiotic resistance genes. However, there is a need for experimental data on production yield, process reproducibility, cost-efficiency, and risk assessment for toxicity to animals and humans and environmental impacts.

## Figures and Tables

**Table 1:** Percent kill of *E. coli* by silver nanoparticle powder from initial to 24 h incubation period.

Treatment (mg AgNP/L)	0 h Incubation	2 h Incubation	4 h Incubation	24 h Incubation
0.625	95.32	98.62	96.18	97.21
1.25	96.31	95.35	95.32	99.53
2.50	99.00	99.35	99.26	99.99
5.00	100	100	100	100

## Data Availability

The original contributions presented in this study are included in the article. Further inquiries can be directed to the corresponding author.

## Abbreviations

The following abbreviations are used in this manuscript:

UV-vis	Ultraviolet-visible spectroscopy
FTIR	Fourier transform infrared
SEM	Scanning electron microscopy
AgNPs	Silver nanoparticles
ATCC	American Type Culture Collection
TSB	Tryptic soy broth
SPR	Surface plasmon resonance
BM	Bitter melon
